# Deep Learning HASTE for Upper Abdominal MRI: Improved Image Quality, Speed, and Energy Efficiency in a Prospective Study

**DOI:** 10.1002/nbm.70183

**Published:** 2025-12-04

**Authors:** Jennifer Gotta, Ahmed Ait Bachir, Leon D. Gruenewald, Ralph Strecker, Dominik Nickel, Simon S. Martin, Christian Booz, Scherwin Mahmoudi, Saber Al‐Saleh, Daniel Dahm, Paul Konrad, Simon Bernatz, Philipp Reschke, Elena Höhne, Renate M. Hammerstingl, Katrin Eichler, Tatjana Gruber‐Rouh, Sebastian M. Haberkorn, Tommaso D'Angelo, Christophe Weber, Stefan Zeuzem, Armin Wiegering, Ralph Sinkus, Thomas J. Vogl, Vitali Koch

**Affiliations:** ^1^ Department of Radiology Goethe University Hospital Frankfurt Frankfurt am Main Germany; ^2^ EMEA Scientific Partnerships Siemens Healthineers AG Forchheim Germany; ^3^ MR Application Predevelopment Siemens Healthineers AG Forchheim Germany; ^4^ Department of Cardiology Goethe University Hospital Frankfurt Frankfurt am Main Germany; ^5^ Department of Biomedical Sciences and Morphological and Functional Imaging University Hospital Messina Messina Italy; ^6^ University Hospital, Department of Cardiology, Angiology, and Pulmonology University of Heidelberg Heidelberg Germany; ^7^ Medical Clinic 1 Goethe University Hospital Frankfurt Frankfurt am Main Germany; ^8^ Department of General, Visceral, and Transplant Surgery Frankfurt University Hospital Frankfurt am Main Germany; ^9^ Laboratory of Translational Vascular Sciences, U1148, INSERM, Université de Paris Paris France

**Keywords:** deep learning, diagnostic imaging, magnetic resonance imaging, neural network

## Abstract

This prospective study aimed to perform a qualitative and quantitative comparison of deep learning (DL) and conventional T2‐weighted Half‐Fourier Acquisition Single‐shot Turbo spin Echo (HASTE) sequences for 3T MRI acquisition of the upper abdomen. From January 2024 to April 2024, 166 patients (60 ± 14 years) scheduled for MRI of the upper abdomen were prospectively enrolled. Each patient underwent two MRI examinations: one using a conventional T2‐weighted HASTE sequence, followed by a fast T2‐weighted HASTE sequence reconstructed with DL. Image quality, anatomical structure visualization, and diagnostic performance were independently assessed by three readers using a 5‐point Likert scale. Quantitative analysis included measurements of signal‐to‐noise ratio (SNR) and contrast‐to‐noise ratio (CNR) for both sequences. Additionally, radiomic features were extracted and analyzed for significant variations. Interreader agreement was evaluated using Fleiss' Kappa. The DL HASTE sequence showed significantly superior overall image quality (*p* < 0.001), fewer artifacts (*p* < 0.001), and improved delineation of anatomical structures (*p* < 0.01) compared to the conventional T2‐weighted HASTE sequence. DL sequences exhibited better SNR (*p* < 0.001), whereas CNR values did not show a difference between the two acquisition types. Radiomics feature analysis unveiled significant differences in contrast and gray‐level characteristics (*p* ≤ 0.001). DL HASTE demonstrated a significant time reduction of 62.5% together with significant energy cost savings of 0.34 kW per scan compared to the conventional sequence acquisition. The DL HASTE sequence enhanced image quality and diagnostic confidence while minimizing artifacts, time, and energy costs, enabling a more accurate detection of pathologies than the conventional T2‐weighted product solution with potential clinical impact.

## Graphical abstract

The DL HASTE sequence enhanced image quality and diagnostic confidence while minimizing artifacts, time, and energy costs, enabling a more accurate detection of pathologies than the conventional T2‐weighted product solution, with direct clinical consequences.

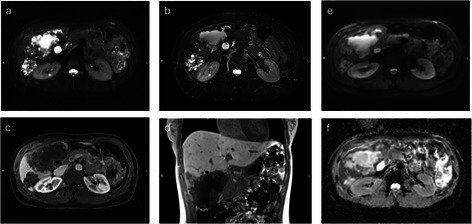

**Abbreviations**
CNR: contrast‐to‐noise ratio
DL: deep learning
HASTE: Half‐Fourier Acquisition Single‐shot Turbo spin Echo
MRI: magnetic resonance imaging
SNR: signal‐to‐noise ratio

## 1 Introduction

Magnetic resonance imaging (MRI) plays a crucial role in the diagnosis and management of various abdominal pathologies [1]. High‐resolution imaging techniques, such as Half‐Fourier Acquisition Single‐shot Turbo spin Echo (HASTE) sequences, are commonly employed for their ability to provide detailed anatomical and pathological information. Traditionally, T2‐weighted HASTE sequences have been used extensively in clinical practice due to their robust imaging capabilities. However, advancements in imaging technology, particularly the advent of deep learning (DL) algorithms, have opened new avenues for enhancing image quality and diagnostic accuracy [2].

DL‐based reconstructions have shown promise in various imaging modalities by improving signal‐to‐noise ratios (SNR), reducing artifacts, and enabling faster acquisition times [3, 4, 5, 6]. These improvements are particularly crucial for abdominal MRI, where image clarity and the ability to discern subtle pathologies can significantly impact patient outcomes. Previous studies on DL‐based reconstructions in abdominal imaging have predominantly been retrospective in design, often limited to smaller or more selected patient cohorts [3, 7]. In contrast, our study prospectively evaluates DL‐reconstructed HASTE sequences in a large, heterogeneous clinical cohort, thereby reflecting real‐world upper abdominal imaging practice. Therefore, this prospective study aimed to perform a qualitative and quantitative comparison of DL HASTE sequences and conventional T2‐weighted HASTE sequences for 3‐Tesla MRI acquisitions of the upper abdomen. The goal was to evaluate the image quality, diagnostic performance, and quantitative metrics such as signal‐to‐noise ratio (SNR) and contrast‐to‐noise ratio (CNR), along with the effectiveness of radiomics features in distinguishing between the two sequences. By addressing these aspects, this study seeks to determine the potential advantages of DL HASTE sequences in clinical practice and their impact on diagnostic confidence and efficiency.

## 2 Methods

Approval for this study was obtained from the local ethics committee. The research was conducted in accordance with their guidelines. All analyses were carried out in compliance with local data protection regulations.

### 2.1 Study Population

This prospective, single‐center study included 166 consecutive patients who were referred for a clinically indicated MRI of the upper abdomen at our institution between January 2024 and May 2024. The study received approval from the local ethics committee and was conducted in full compliance with local data protection regulations. Participation in the study was voluntary, and all patients provided written informed consent to undergo additional imaging during the same examination. The research DL acquisition MRI sequence was provided by Siemens Healthineers (Forchheim, Germany). The authors, who have no employment ties with Siemens Healthineers, were the only ones with access to the patient data.

Inclusion criteria were age over 18 years and a clinically scheduled MRI of the upper abdomen for any clinical indication. The exclusion criteria included the inability to undergo MRI (such as claustrophobia, metallic implants, or pregnancy), as well as those who did not consent to participate. Patients with incomplete MRI datasets were also excluded from the study. The acquisition order was fixed, with the conventional T2‐weighted HASTE sequence performed first, followed by the DL HASTE sequence; patients were carefully coached, and repeated acquisitions were obtained when breath‐hold failure or motion artifacts were observed. Both conventional and DL HASTE sequences were acquired during a single breath‐hold with verbal coaching; typical breath‐hold durations were 15 s. Energy consumption was estimated from vendor‐provided specifications of sequence‐specific power usage in combination with recorded scan durations, rather than measured directly at the scanner.

### 2.2 DL Reconstruction Technique

As a single‐shot acquisition, HASTE is on the one hand limited by low SNR and on the other hand limited by T2 blurring due to longer echo trains compared to segmented acquisitions. The main target of the DL‐enhanced HASTE is to allow for higher undersampling factors to shorten the echo train and to decrease the intrinsic T2 blurring. Indirectly, this also leads to a reduction in the specific absorption rate (SAR) as fewer refocusing pulses are applied and consequently allows shortening the acquisition time by lowering the repetition time (Table S1).

The DL‐based image reconstruction is inspired by variational networks and based on a recently explored variant [7, 8]. The network architecture receives undersampled k‐space data and precalculated coil sensitivity maps as input and generates images iteratively by data consistency updates based on a parallel imaging model that is later interleaved with network‐based image enhancements. The first 22 iterations are performed with data consistency updates only, whereas the following 11 iterations include network‐based image enhancement. Trainable parameters comprised the step sizes of the data consistency updates and the model parameters of the individual image enhancement networks.

The complete architecture was trained in a supervised manner using about 10,000 slices obtained from healthy volunteers on various clinical 1.5T and 3T scanners (MAGNETOM, Siemens Healthineers, Erlangen, Germany). The training data were acquired using conventional HASTE protocols with a parallel imaging acceleration of 2, and ground truth was obtained using parallel imaging. Input to the reconstruction network was retrospectively downsampled to an acceleration factor of 4. The training was implemented in PyTorch and performed on a NVIDIA Tesla V100 GPU. The obtained model parameters were then exported for prospective use on a clinical MRI scanner within a research application.

### 2.3 Image Analysis

Three readers (from our institution) with 4–12 years of experience in abdominal imaging conducted an independent image analysis. Each reader assessed the standard T2‐weighted HASTE and the corresponding T2‐weighted HASTE using DL reconstruction in separate sessions to minimize recall bias. The readers were blinded to each other's evaluations. Image analysis was conducted on a dedicated workstation (GE Centricity PACS RA1000; GE Healthcare). Various outcome measures were assessed, including the homogeneity of fat suppression, image noise, lesion sharpness, artifact level, diagnostic confidence, and overall image quality. The readers used a 5‐point Likert scale to evaluate these measures, where 1 indicated the *poorest quality*, 2 indicated *poor*, 3 indicated *moderate*, 4 indicated *good*, and 5 indicated *excellent*. A higher score reflects better image quality, including more effective fat suppression, higher SNR, improved image contrast, sharper edges, better lesion detectability, lower artifact levels, and greater diagnostic confidence.

### 2.4 Radiomics Analysis

For radiomics analysis, the MRI datasets of each patient were extracted in Digital Imaging and Communications in Medicine (DICOM) format and uploaded into 3D Slicer (Version 5.0.2, Harvard University, Cambridge, United States). A region of interest (ROI) was then placed in the liver parenchyma of the right hepatic lobe, while avoiding vessels and any pathological areas in both conventional and DL sequences. An experienced radiologist reviewed each segmentation. If any discrepancies were noted in the initial segmentation, adjustments were made accordingly following a repeated segmentation process.

Using the PyRadiomics extension within 3D Slicer (Version 5.1.0‐2022‐05‐20), we extracted 107 radiomics features from each segmentation (Table S2) [9]. These features were then organized into seven categories: Gray‐Level Dependence Matrix (GLDM), Gray‐Level Cooccurrence Matrix (GLCM), Gray‐Level Run Length Matrix (GLRLM), Gray‐Level Size Zone Matrix (GLSZM), Neighboring Gray Tone Difference Matrix (NGTDM), Shape, and First Order [9].

### 2.5 SNR and CNR Measurements

For quantitative image analysis, SNR and CNR were calculated. Two circular regions of interest (ROIs), each with an area of 1.5 cm^2^, were placed in a healthy part of the right hepatic lobe, carefully excluding hepatic vessels, lesions, artifacts (such as repetition and metallic artifacts), and sudden signal changes, in both the standard T2‐weighted HASTE and the DL‐T2‐weighted HASTE sequences. Additionally, an ROI was placed in the background to measure the noise level. SNR quantifies the ratio between the signal strength and background noise and was computed using the following formula:

$$\mathrm{SNR}=\frac{\mathrm{Avg}\left(\text{liver}1\right)-\mathrm{Avg}\left(\text{background}\right)}{\mathrm{Std}\left(\text{background}\right)}.$$

For the DL‐enhanced method, the calculation was adjusted accordingly:

$${\mathrm{SNR}}_{\mathrm{DL}}=\frac{\mathrm{Avg}\left(\text{liver}1_{\mathrm{DL}}\right)-\mathrm{Avg}\left({\text{background}}_{\mathrm{DL}}\right)}{\mathrm{Std}\left({\text{background}}_{\mathrm{DL}}\right)}.$$

The CNR measures the contrast between two signal regions relative to the noise and was calculated as follows:

$$\mathrm{CNR}=\frac{\mathrm{Avg}\left(\text{liver}1\right)-\mathrm{Avg}\left(\text{liver}2\right)}{\mathrm{Std}\left(\text{background}\right)}.$$

For the DL‐enhanced method, the calculation was similarly adjusted:

$${\mathrm{CNR}}_{\mathrm{DL}}=\frac{\mathrm{Avg}\left(\text{liver}1_{\mathrm{DL}}\right)-\mathrm{Avg}\left(\text{liver}2_{\mathrm{DL}}\right)}{\mathrm{Std}\left({\text{background}}_{\mathrm{DL}}\right)}.$$

The average values (*Avg*) of the signal regions (e.g., *liver*1 and *liver*2) and the standard deviations (Std) of the background noise were extracted from the image data to compute the SNR and CNR values for both methods, facilitating their comparison.

### 2.6 Statistical Analysis

Statistical analysis was performed using R statistical software (R Foundation for Statistical Computing, Vienna, Austria; Version 2023.06.0 + 421) and MedCalc (MedCalc Software Ltd., Ostend, Belgium; Version 20.123). Normally distributed data were presented as mean ± standard deviation, whereas nonnormally distributed data were reported as median and interquartile range (IQR).

An independent samples *t* test was conducted to compare the means of normally distributed variables between the standard T2‐weighted HASTE and the T2‐weighted HASTE using DL reconstruction. To verify the normality of the data, the Shapiro–Wilk test and the D'Agostino–Pearson test were employed. When the data were found to be nonnormally distributed, the Mann–Whitney *U* test was used as an alternative. Interreader agreement was evaluated using Fleiss' Kappa [10] with the following interpretation: 0.00–0.20 = slight; 0.21–0.40 = fair; 0.41–0.60 = moderate; 0.61–0.80 = substantial; and 0.81–1.00 = almost perfect.

## 3 Results

### 3.1 Patient Characteristics

**FIGURE 1:**
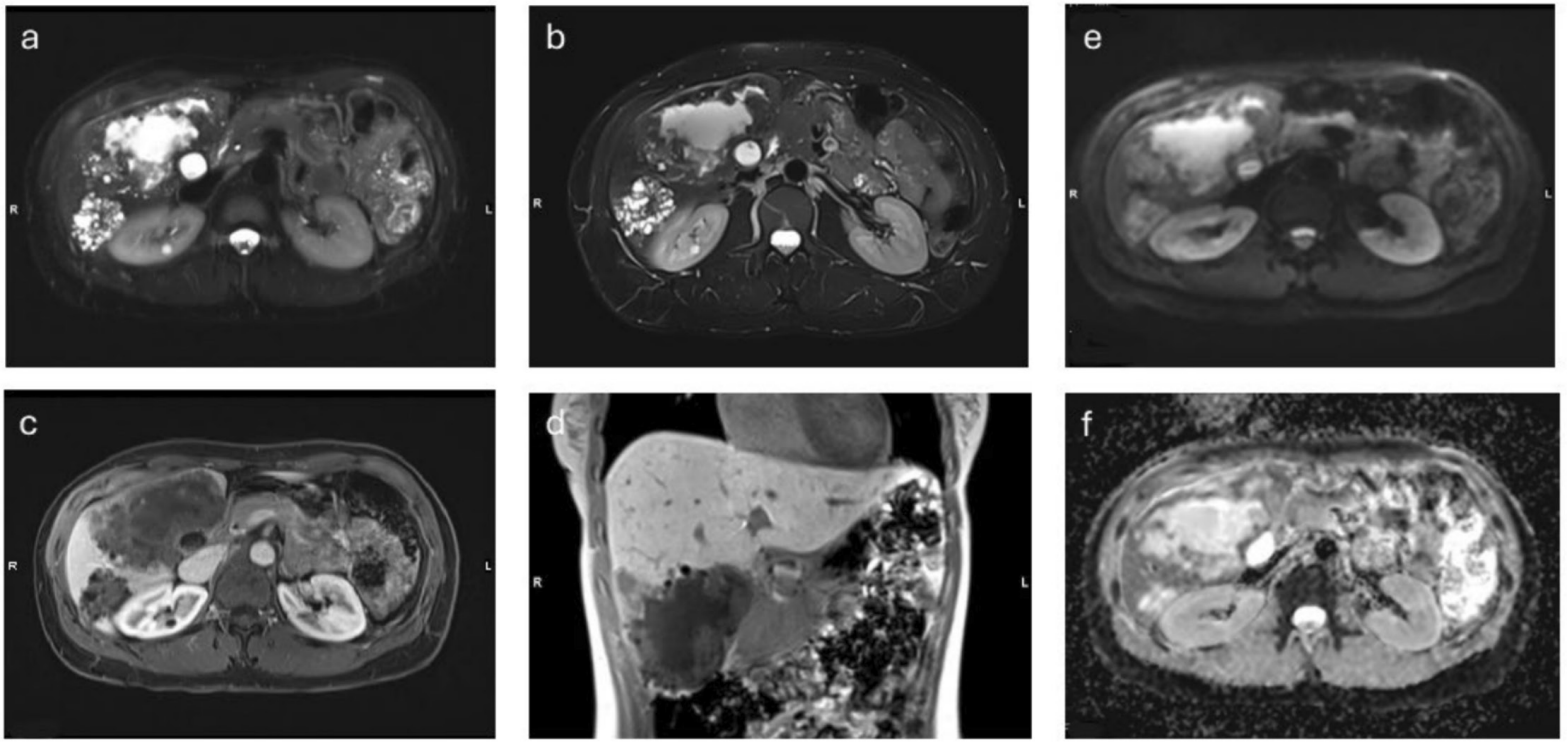
MRI of a 46‐year‐old female with a palpable mass of the upper right abdomen showing inhomogeneous liver parenchyma with multiple cystic lesions in segments 4a/b and 5–7 and multiple smallnodular foci. (a) Axial, noncontrast conventional HASTE sequence; (b) axial, noncontrast DL‐HASTE sequence; (c) contrast‐enhanced T1‐sequence (venous phase); (d) coronal contrast‐enhanced T1 (venous phase); (e) diffusion map (TRACE); and (f) diffusion map (ADC).

**FIGURE 2:**
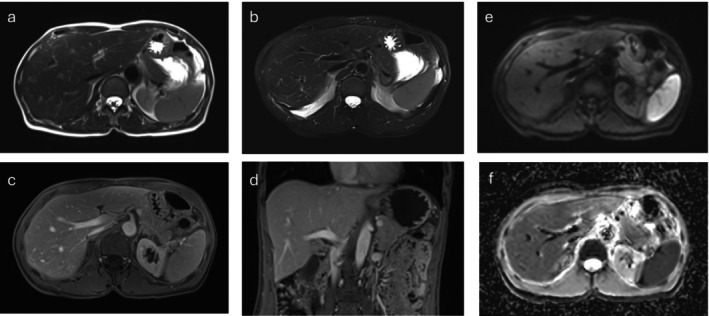
MRI of a 56‐year‐old female with suspected Morbus Crohn of Ileum. (a) Axial T2‐HASTE sequence, (b) axial DL‐HASTE sequence, (c) axial T1 sequence, (d) coronal T1 sequence, (e) diffusion map (Trace), and (f) diffusion map (ADC).

A total of 166 patients, with a mean age of 60 ± 14 years, were included in the study. The age distribution ranged from 21 to 84 years. Among these, 102 were male (61%), and 64 were female (39%). The most frequent primary diagnoses included liver metastases from breast cancer (25%), pancreatic carcinoma (15%), desmoid fibroma (10%), and liver metastases from duodenal carcinoma (10%). Hematological parameters revealed a mean hemoglobin level of 13.2 ± 1.8 g/dL and a mean leukocyte count of 7.4 ± 2.1 × 10^9^/L. The mean platelet count was 185.3 ± 96.2 × 10^9^/L. Table 1 presents a comprehensive summary of the sociodemographic and clinical characteristics. Figures 1 and 2 are examples of conventional T2‐weighted HASTE and T2‐weighted HASTE using DL reconstruction.

**TABLE 1:** Baseline characteristics of the study population.

Variables	Full sample (*N* = 166)
*n* (%) or mean (SD) or median (IQR)	
Demographics	
Overall age (years)	60 ± 14
Male sex (*n*, %)	102 (61%)
Female sex (*n*, %)	64 (39%)
BMI (kg/m^2^)	24.6 ± 4.6
Laboratory parameters	
GPT (U/L)	45 ± 52
GOT (U/L)	50 ± 47
*γ*GT (U/L)	145 ± 236
Alcalic phosphatase (U/l)	153 ± 125
LDH (U/L)	302 ± 621
INR	1.1 ± 0.2
Thrombocytes (/nL)	185 ± 96
Primary diagnosis	
Liver metastasis (*n*)	47 (28%)
HCC (*n*)	24 (14%)
CCC (*n*)	8 (5%)
Pancreas carcinoma (*n*)	5 (3%)
Liver cirrhosis (*n*)	3 (2%)
Colorectal carcinoma (*n*)	2 (1%)
Others (n)	77 (46%)

Abbreviations: *γ*GT, gamma‐glutamyl‐transferase; BMI, body mass index; CCC, cholangiocarcinoma; GOT, glutamat‐oxalacetat‐transaminase; GPT, glutamat‐pyruvat‐transaminase; HCC, hepatocellular carcinoma; INR, international normalized ratio; IQR, interquartile range; LDH, lactatdehydrogenase; SD, standard deviation.

### 3.2 Image Quality

The mean diagnostic confidence was 3.9 ± 0.7 for standard T2‐weighted HASTE and 4.6 ± 0.6 for DL HASTE (*p* < 0.001).

Overall image quality was 3.9 ± 0.7 for standard T2‐weighted HASTE and 4.5 ± 0.5 for DL HASTE (*p* < 0.001). The delineation of anatomical structures was significantly higher with the DL HASTE sequence (*p* < 0.001). The presence of artifacts was considerably lower for the DL HASTE sequence than for the T2‐weighted HASTE sequence (*p* < 0.001).

The SNR values for the liver parenchyma were significantly better for DL HASTE compared to standard T2‐weighted HASTE. The median SNR of the standard T2‐weighted HASTE was 48.8 (IQR, 0.4–270), whereas the median SNR of the DL HASTE was 115 (IQR, −62.5 to 285) (*p* < 0.001).

**FIGURE 3:**
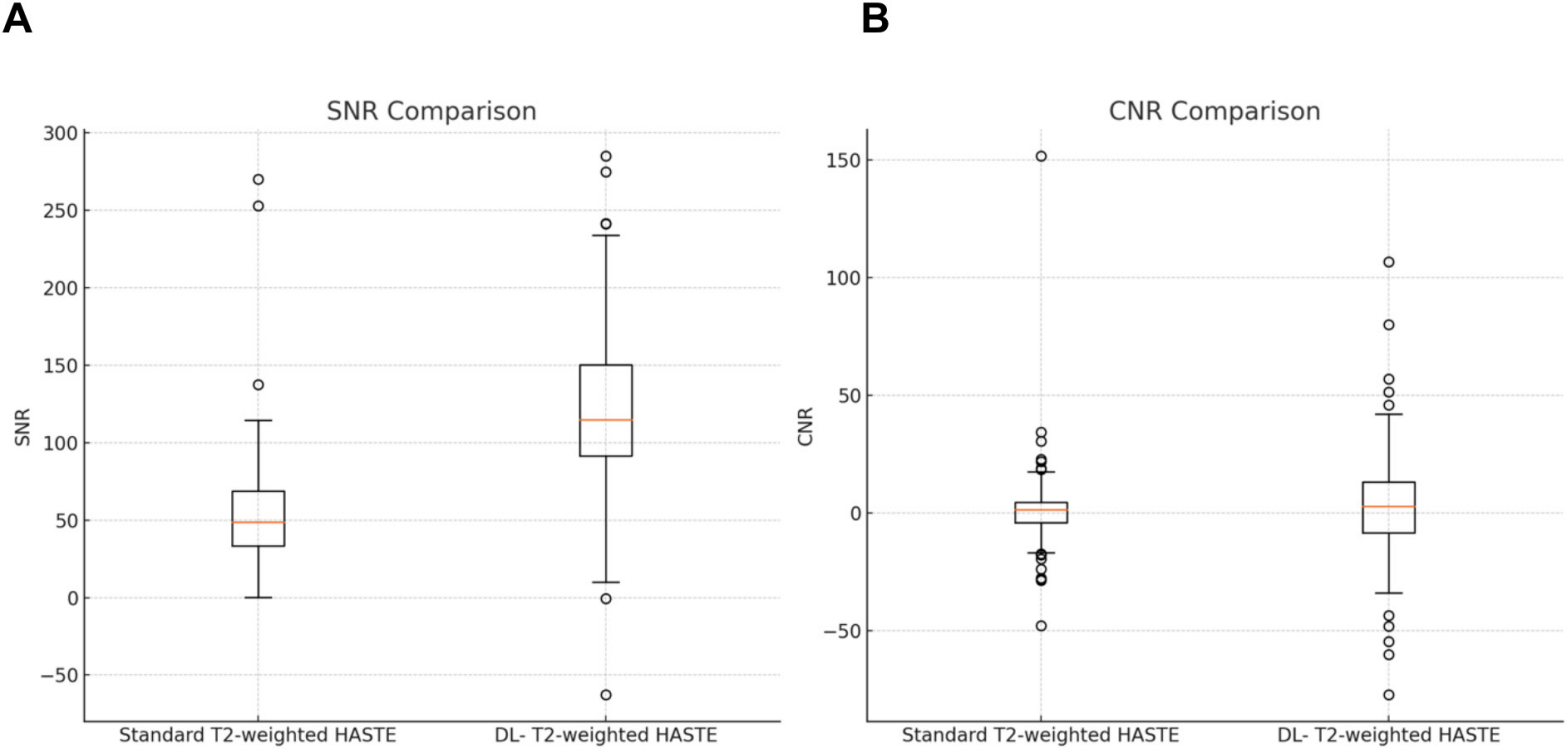
Boxplot of SNR and CNR metrics. This boxplot displays the distribution of SNR (A) and CNR (B) metrics of the standard T2‐weighted HASTE sequence and the DL‐T2‐weighted HASTE sequence. Abbreviations: CNR, contrast‐to‐noise ratio; SNR, signal‐to‐noise ratio.

In contrast, the CNR values did not significantly differ between the two sequences. The median CNR of the standard T2‐weighted HASTE was 1.50 (IQR, −47.65 to 151.54), and the median CNR of the DL‐ HASTE was 3.00 (IQR, −77.0 to 106.67), with a Wilcoxon signed‐rank test indicating no significant difference (*p* = 0.228). Detailed results are presented in Figure 3.

Furthermore, the T2‐weighted HASTE sequence demonstrated a significant reduction in duration, with a time reduction of 62.5% with DL HASTE compared to conventional methods (0.18 vs. 0.48 min; *p* < 0.05).

### 3.3 Interreader Agreement

The interreader agreement ranged from moderate to almost perfect, with values between 0.47 and 0.97 for standard T2‐weighted HASTE and between 0.61 and 0.86 for DL HASTE, as illustrated in Table 2.

**TABLE 2:** Image quality of standard T2‐weighted HASTE and DL‐accelerated T2‐weighted HASTE sequences.

Parameter	Standard T2‐weighted HASTE		DL‐T2‐weighted HASTE		*p* value
	Rating	Agreement	Rating	Agreement	
Image quality	3.9 ± 0.7	0.47	4.5 ± 0.5	0.82	< 0.01
Image noise	3.8 ± 0.7	0.97	4.5 ± 0.5	0.83	< 0.01
Artifacts	3.8 ± 0.7	0.92	4.5 ± 0.5	0.73	< 0.01
Fat suppression	3.8 ± 0.8	0.86	4.5 ± 0.6	0.85	< 0.01
Lesion sharpness	3.4 ± 0.7	0.88	4.6 ± 0.5	0.78	< 0.01
Diagnostic confidence	3.9 ± 0.7	0.84	4.6 ± 0.6	0.86	< 0.01
Delineation of anatomical structures					
Liver	3.8 ± 0.8	0.87	4.5 ± 0.6	0.84	< 0.01
Pancreas	3.4 ± 0.7	0.87	4.4 ± 0.6	0.82	< 0.01
Spleen	3.9 ± 0.7	0.86	4.4 ± 0.6	0.61	< 0.05
Kidney	3.9 ± 0.8	0.87	4.5 ± 0.6	0.85	< 0.01
Heart	4.0 ± 0.8	0.89	4.5 ± 0.6	0.82	< 0.01

Abbreviations: DL, deep learning; HASTE, Half‐Fourier Single‐shot Turbo spin‐Echo.

### 3.4 Radiomics Analysis

In this study, radiomics features extracted from two different imaging sequences—conventional and DL‐based HASTE sequences—were compared to identify significant differences in texture and gray‐level properties. A total of 20 radiomics features showed statistically significant differences (*p* < 0.05). The most significant features were from the category of Gray Level Dependence Matrix (GLDM), which quantifies gray level dependencies in an image: gldm_DependenceNonUniformity (*p* < 0.001) and gldm_GrayLevelNonUniformity (*p* < 0.001) (Figure S1).

### 3.5 Energy Efficiency of Deep‐Learning‐Enhanced MRI Sequences

The conventional sequence exhibited an energy consumption of 0.54 kW per examination, whereas the DL‐enhanced sequence required only 0.20 kW, resulting in a reduction of 0.34 kW per scan. For a typical daily workload of 100 examinations, this translates to an energy saving of 34 kW per day, equivalent to 12,410 kW annually. Assuming an average electricity cost of 0.30 €/kWh, the financial savings amount to approximately 3723 € per year.

## 4 Discussion

Our study prospectively assessed the use of a DL reconstruction method for upper abdominal MRI in routine clinical practice. The aim was to evaluate and compare the performance of DL HASTE sequences with conventional T2‐weighted HASTE sequences for upper abdominal MRI at 3 Tesla.

Compared to conventional T2‐weighted HASTE sequences, the DL HASTE sequences significantly improved quantitative image features, such as SNR (*p* < 0.001), and overall image quality while maintaining diagnostic confidence. These results suggest that DL‐enhanced sequences could offer substantial advantages in clinical imaging, particularly for patients requiring high‐quality imaging to detect and characterize abdominal pathologies.

The significantly higher SNR values observed in DL‐HASTE sequences (median SNR: 115.00 vs. 48.75, *p* < 0.001) indicate superior image clarity, essential for accurately identifying small or subtle lesions, for example, within the liver parenchyma. In clinical practice, this improvement could enhance lesion conspicuity and reduce the likelihood of false negatives, ultimately supporting better clinical decision‐making. Interestingly, the CNR values did not show a significant difference between the two sequences, suggesting that although DL reconstruction enhances signal intensity, it does not compromise contrast differentiation between adjacent tissues. In contrast, a previous study demonstrated that a different approach to HASTE image acquisition provided superior image quality and higher CNR than conventional HASTE sequences [11]. This finding is consistent with prior research, showing that DL algorithms can optimize image quality, reduce artifacts, and enhance lesion visibility when using DL HASTE sequences [12].

The reduction in acquisition time by 62.5% using DL‐HASTE sequences is another advantage, as it minimizes patient discomfort and susceptibility to motion artifacts, common in abdominal MRI. The ability to acquire high‐quality images in a shorter time frame is particularly relevant for routine clinical practice. This improves patient comfort and increases throughput, which is especially beneficial for patients with limited tolerance for prolonged scans, such as pediatric or claustrophobic patients. Furthermore, the reduced scan duration has led to fewer repeat scans and a significant decrease in wait times due to faster scan turnover.

Additionally, the higher quality images facilitate more accurate and earlier diagnoses, enabling timely detection and treatment, which is especially valuable for cancer patients. Furthermore, DL‐enhanced sequences not only enhance imaging quality but also significantly alleviate the environmental and economic impact of routine MRI examinations. The marked reduction in energy consumption underscores the transformative potential of innovative technologies in promoting sustainable and efficient practices within radiology.

Moreover, our analysis demonstrated that DL‐HASTE sequences resulted in significantly lower artifact levels, particularly motion artifacts, which can degrade image quality and affect diagnostic accuracy [13, 14]. From a radiomics perspective, the DL‐HASTE sequences exhibited distinct texture and gray‐level properties compared to conventional HASTE, with 20 radiomic features showing statistically significant differences (*p* < 0.05). The most prominent features were GLDM‐based metrics, such as dependence nonuniformity and gray‐level nonuniformity, highlighting the capacity of DL‐based sequences to capture complex tissue patterns despite reduced acquisition time and potential raw data loss.

However, our study has several limitations that should be considered. First, it was conducted at a single center to minimize variability associated with scanners from different manufacturers or generations. This, combined with the limited sample size, may affect the generalizability of our findings. Second, despite using standardized evaluation criteria, the subjective interpretation by individual readers is an inherent aspect of assessing image quality parameters. Variability in reader experience and expertise could introduce bias or inconsistencies. A low interreader agreement can arise from various factors linked to differences in image interpretation, methodological aspects, and individual reader experience. Third, potential variations in patient physiology, such as different breathing patterns and patient positioning, may have influenced the results.

Another limitation of our study is that SNR and CNR measurements were based on two relatively small circular ROIs (~1.5 cm^2^) in the right hepatic lobe, which may be susceptible to B1 inhomogeneity and local texture variations; alternative strategies such as larger or multiple ROIs could further enhance robustness in future studies.

Despite the promising results of DL‐based HASTE reconstruction, several practical aspects must be considered before broad clinical implementation. First, the availability of this technology currently depends on vendor‐specific hardware and software solutions, which may limit accessibility across institutions. Second, regulatory clearance and compliance with local data protection standards are required before routine deployment, potentially delaying widespread adoption. Third, radiologists and technicians need to become familiar with the image characteristics of DL‐reconstructed sequences, which may differ slightly from conventional images and therefore necessitate dedicated training. Over time, increasing availability, technical standardization, and regulatory approval are expected to facilitate integration into routine workflows, but these factors should be acknowledged as current limitations.

Another limitation of our energy analysis is that consumption was estimated based on scanner specifications and scan time rather than direct measurements, which may not capture patient or site‐specific variations in power demand.

Additionally, our study did not include external validation using phantoms. Although the focus was on prospective in vivo patient data to evaluate clinical feasibility, future work should incorporate phantom‐based validation to confirm reproducibility and technical stability of the DL reconstruction.

Furthermore, interreader and intrareader variability of ROI placement was not formally assessed, which may have introduced bias despite expert review and correction of segmentations.

In summary, DL HASTE sequences offer superior image quality, reduced artifacts, and shorter acquisition times than conventional HASTE sequences, making them a valuable addition to routine abdominal MRI. The distinct radiomic feature profiles associated with DL‐HASTE sequences highlight their potential for advanced tissue characterization and improved diagnostic accuracy. These results advocate for the expanded implementation of DL‐enhanced imaging techniques in clinical practice, as they can potentially improve diagnostic workflows and optimize patient outcomes.

## Author Contributions

All authors have contributed significantly to this work, participating in the conception, design, analysis, interpretation of data, and writing/editing of this manuscript. J.G. wrote the manuscript and V.K. designed the research. The other authors were involved in the analysis of the data and performed research or assisted with the technical development. All authors revised the manuscript.

## Funding

This study was supported by the Wilhelm Vaillant Stiftung (82700741).

## Ethics Statement

Institutional Review Board approval was obtained.

## Conflicts of Interest

C.B. received speaking fees from Siemens Healthineers. V.K. received travel support from Siemens Healthineers.

## Supporting information


**Table S1:** MRI acquisition parameters.
**Table S2:** Radiomics features with corresponding feature classes.
**Figure S1:** Bar plot illustrating the significant radiomics features. Abbreviations: GLCM, gray‐level cooccurrence matrix; GLRLM, gray‐level run length matrix; GLSZM, gray‐level size zone matrix; NGTDM, neighboring gray tone difference matrix; GLDM, gray‐level dependence matrix.

## Data Availability

The data of this study are available from the corresponding author upon reasonable request.
