# Supplementary material for: Deep Learning HASTE for Upper Abdominal MRI: Improved Image Quality, Speed, and Energy Efficiency in a Prospective Study
Source: NMR Biomed. 2025 Dec 4;39(1):e70183. doi: 10.1002/nbm.70183 (PMC12678876; doi:10.1002/nbm.70183)
Supplement: Supplementary file 1 — Table S1: MRI acquisition parameters. Table S2: Radiomics features with corresponding feature classes. Figure S1: Bar plot illustrating the significant radiomics features. Abbreviations: GLCM, gray‐level cooccurrence matrix; GLRLM, gray‐level run length matrix; GLSZM, gray‐level size zone matrix; NGTDM, neighboring gray tone difference matrix; GLDM, gray‐level dependence matrix. [file NBM-39-e70183-s001.docx]

**Supplementary Table**

**Table S1:** MRI acquisition parameters.

| **Acquisition parameter** | **Conventional HASTE** | **DL HASTE** |
| --- | --- | --- |
| Orientation | Axial | Axial |
| Acquisition type | 2D | 2D |
| Echo time [ms] | 93 | 95 |
| Flip angle [degree] | 130 | 113 |
| Repetition time [ms] | 1060 | 500 |

Abbreviations: DL = deep learning; MRI = magnetic resonance imaging.

**Supplementary Table S2.** Radiomics features with corresponding feature classes.

| **Feature class** | **Radiomics features** |  |
| --- | --- | --- |
| Shape | Mesh volume, voxel volume, voxel number, surface area, surface volume ratio, sphericity, compactness, spherical disproportion, maximum 3D diameter, maximum 2D diameter (slice), maximum 2D diameter (column), maximum 2D diameter (row), major axis length, minor axis length, least axis length, elongation, flatness |  |
| First order | Energy, total energy, entropy, minimum, 10th percentile, 90th percentile, maximum, mean, median, interquartile range, range, mean absolute deviation (MAD), robust mean absolute deviation (rMAD), root mean square (RMS), standard deviation, skewness, kurtosis, variance, uniformity |  |
| GLCM | Autocorrelation, joint average, cluster prominence, cluster shade, cluster tendency, contrast, correlation, difference average, difference entropy, difference variance, joint energy, joint entropy, informational measure of correlation (IMC) 1, informational measure of correlation (IMC) 2, inverse difference moment (IDM), maximal correlation coefficient (MCC), inverse difference moment normalized (IDMN), inverse difference (ID), inverse difference normalized (IDN), inverse variance, maximum probability, sum average, sum entropy, sum of squares |  |
| GLDM | Small dependence emphasis (SDE), large dependence emphasis (LDE), gray-level non-uniformity (GLN), dependence non-uniformity (DN), dependence non-uniformity normalized (DNN), gray-level variance (GLV), dependence variance (DV), dependence entropy (DE), low gray-level emphasis (LGLE), high gray-level emphasis (HGLE), small dependence low gray-level emphasis (SDLGLE), small dependence high gray-level emphasis (SDHGLE), large dependence low gray-level emphasis (LDLGLE), large dependence high gray-level emphasis (LDHGLE) |  |
| GLRLM | Short-run emphasis (SRE), long-run emphasis (LRE), gray-level non-uniformity (GLN), gray-level non-uniformity normalized (GLNN), run length non-uniformity (RLN), run length non-uniformity normalized (RLNN), run percentage (RP), gray-level variance (GLV), run variance (RV), run entropy (RE), low gray-level run emphasis (LGLRE), high gray-level run emphasis (HGLRE), short-run low gray-level emphasis (SRLGLE), short-run high gray-level emphasis (SRHGLE), long-run low gray-level emphasis (LRLGLE), long-run high gray-level emphasis (LRHGLE) |  |
| GLSZM | Small-area emphasis (SAE), large-area emphasis (LAE), gray-level non-uniformity (GLN), gray-level non-uniformity normalized (GLNN), size-zone non-uniformity (SZN), size-zone non-uniformity normalized (SZNN), zone percentage (ZP), gray-level variance (GLV), zone variance (ZV), zone entropy (ZE), low gray-level zone emphasis (LGLZE), high gray-level zone emphasis (HGLZE), small-area low gray-level emphasis (SALGLE), small-area high gray-level emphasis (SAHGLE), large-area low gray-level emphasis (LALGLE), large-area high gray-level emphasis (LAHGLE) |  |
| NGTDM | Coarseness, contrast, busyness, complexity, strength |  |

Abbreviations: GLCM, gray-level cooccurrence matrix; GLRLM, gray-level run length matrix; GLSZM, gray-level size zone matrix; NGTDM, neighboring gray tone difference matrix; GLDM, gray-level dependence matrix.

**Supplemental Figure 1**


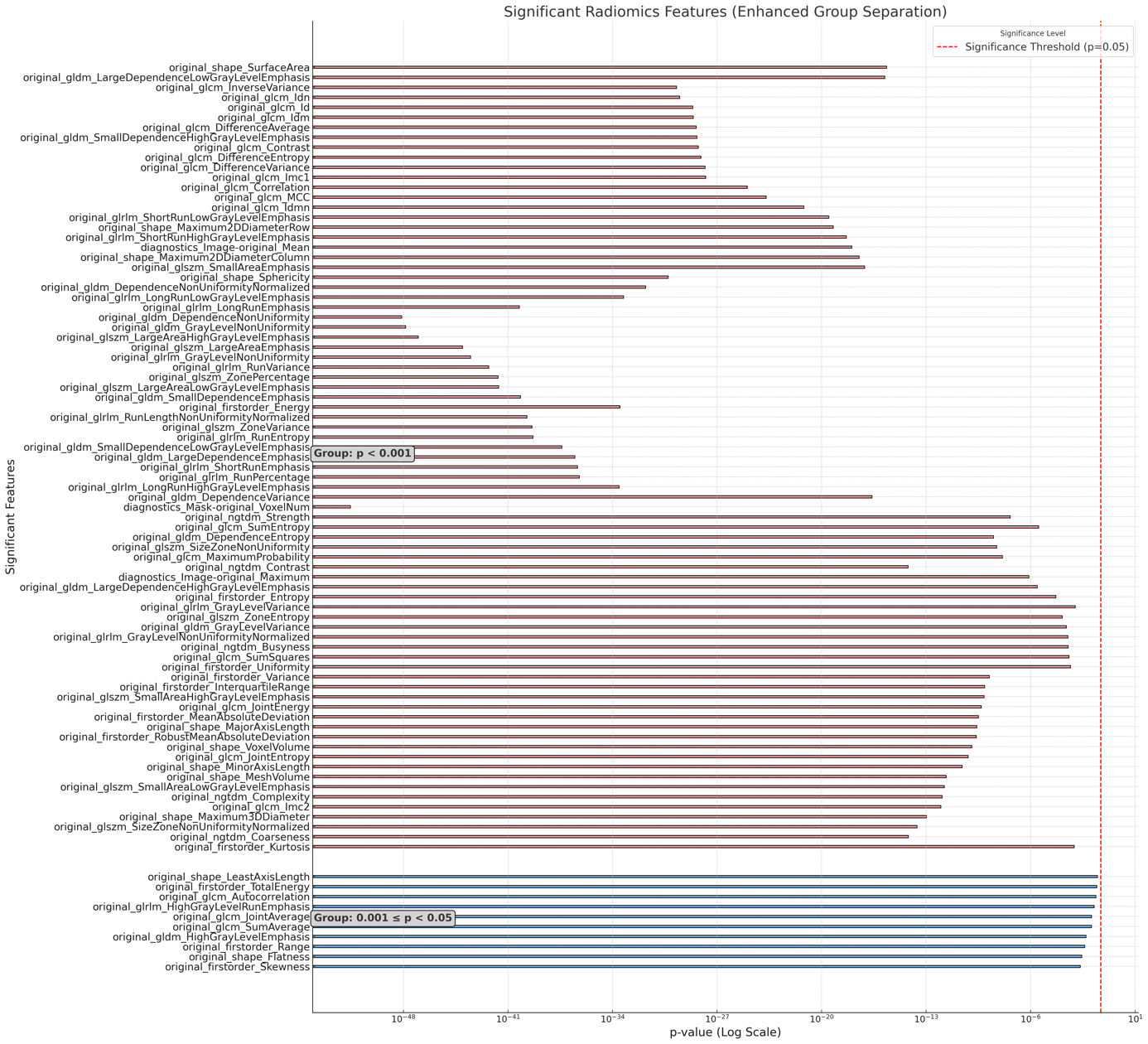


Bar plot illustrating the significant radiomics features.

Abbreviations: GLCM, gray-level cooccurrence matrix; GLRLM, gray-level run length matrix; GLSZM, gray-level size zone matrix; NGTDM, neighboring gray tone difference matrix; GLDM, gray-level dependence matrix.
